# Rectal Mucosal Prolapse Syndrome: MRI and Endoscopic Images Mimicking Crohn’s Disease

**DOI:** 10.3390/diagnostics15192417

**Published:** 2025-09-23

**Authors:** Zhaoyang Li, Meng Sun, Yufang Wang

**Affiliations:** Department of Gastroenterology and Hepatology, West China Hospital, Sichuan University, Chengdu 610041, China; li_zy2000@163.com (Z.L.);

**Keywords:** rectal mucosal prolapse syndrome, Crohn’s disease, misdiagnosis, MRI, endoscopy, mimic

## Abstract

Rectal mucosal prolapse syndrome (RMPS) is a rare condition that mimics Crohn’s disease (CD) in imaging and endoscopy. We present a 29-year-old male with rectal tenesmus and a mid-rectal mass initially diagnosed as CD based on colonoscopy and MRI. After failed corticosteroid therapy, endoscopic resection revealed fibromuscular obliteration pathognomonic for RMPS. Chronic prolapse may induce secondary inflammation mimicking CD (including ulceration, stricture, and pseudofistulation), necessitating careful differential diagnosis to avoid unwarranted corticosteroid therapy.

**Figure 1:**
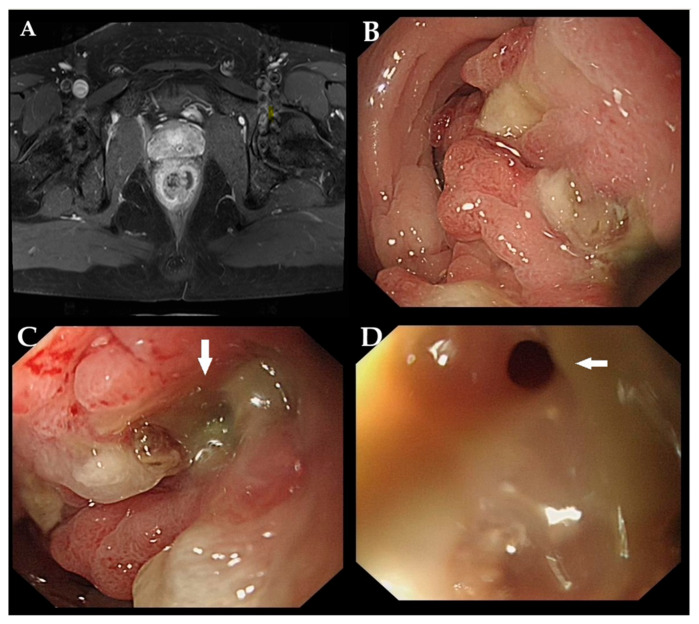
A 29-year-old male was admitted to the hospital for chronic rectal tenesmus and constipation. Digital rectal examination revealed an immovable and hard mass located 5 cm from the anal verge. Blood tests revealed elevated levels of C-reactive protein and immunoglobulin E. Magnetic resonance imaging revealed circumferential wall thickening with a mass-like lesion in the mid-rectum (**A**). Colonoscopy showed an irregular nodular lesion at 5 cm from the anal verge with scant purulent discharge adherent to the surface, leading to rectal stricture (**B**). A mucus-discharging fistula was opening at the center of the lesion ((**C**,**D**) white arrow). Initial biopsies revealed chronic inflammation of the mucosa, accompanied by hyperplastic glandular epithelial cells. Based on these findings, a diagnosis of Crohn’s disease (CD) was suspected.

**Figure 2:**
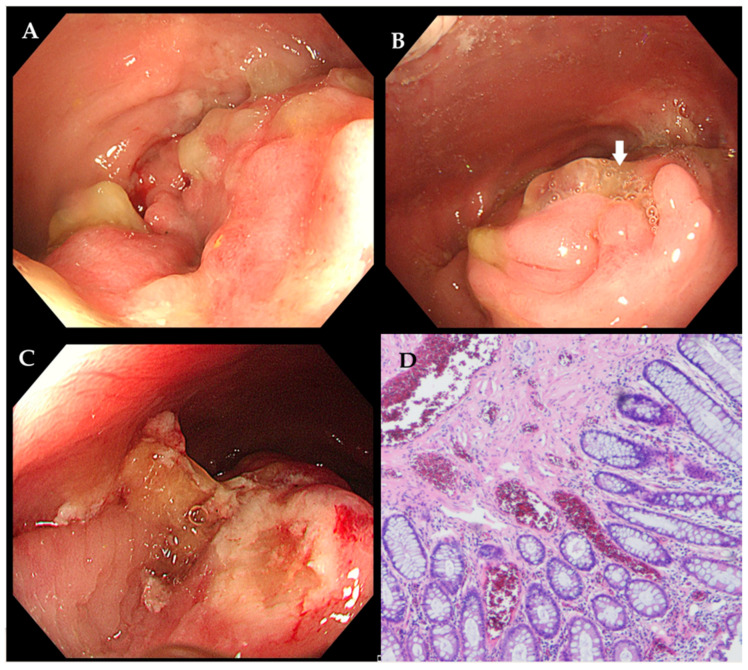
Due to the recurrence of symptoms after a brief response to a 5-month corticosteroid trial, endoscopic mucosal resection was performed, revealing a persistent lesion with fistulation ((**A**,**B**) indicated by the white arrow). The lesion was resected (**C**). Histology confirmed fibromuscular obliteration of the lamina propria and smooth muscle hyperplasia between crypts ((**D**) hematoxylin and eosin staining, ×40)—findings inconsistent with CD. The patient first underwent conservative treatment for three months to adjust bowel habits and relieve constipation, but the symptoms did not improve significantly. Subsequently, the patient underwent a series of surgical interventions, including high-level complex anal fistula excision, incision and drainage of a submucosal rectal abscess, and laparoscopic transanal intersphincteric resection. Intraoperative exploration revealed a circumferential inflammatory mass protruding into the rectal lumen. The submucosa was filled with mucoid tissue and inflammatory necrotic tissue, which had a hard consistency. Additionally, a complex high-level anal fistula with three internal openings was formed within the range of 5 cm to 9 cm from the dentate line. Examination of the resected specimen showed a circumferential mass with a cobblestoned mucosal appearance and deep ulceration. The diagnosis of RMPS was again confirmed histologically by fibromuscular hyperplasia and submucosal hyalinization, which excluded CD. After a long-term follow-up of two and a half years, the patient remains asymptomatic, with no recurrence of symptoms. RMPS is a rare but clinically significant condition that can closely mimic CD in both imaging and endoscopic findings, leading to potential misdiagnosis and inappropriate treatment. While both conditions may exhibit mucosal ulceration, strictures, and pseudofistulas, RMPS typically lacks transmural inflammation, granulomas, or mesenteric involvement, which are hallmarks of CD [1]. Moreover, endoscopic mucosal resection (EMR) with thorough histopathological evaluation is essential when clinical and imaging findings are ambiguous, as RMPS exhibits distinct fibromuscular proliferation without chronic inflammation [2]. This case underscores the importance of a multidisciplinary approach in preventing unnecessary immunosuppression and ensuring optimal surgical intervention. In daily practice, a high index of suspicion for RMPS is the crucial first step when evaluating puzzling or refractory “CD-like” rectal lesions, particularly when atypical features are present [3]. Securing a definitive diagnosis requires deep and repeated biopsies to obtain adequate submucosal tissue. Finally, a multidisciplinary team discussion is essential whenever a clear discrepancy exists between the clinical, endoscopic, or radiological findings and the pathological results. Additionally, Zhou et al. reported a case of RMPS presenting as diffuse polyposis and colitis cystica profunda in a teenage boy suffering from refractory fibrostenotic CD [4]. It is important to note that RMPS may coexist with IBD, where the diagnosis becomes particularly challenging, and traditional therapies may not be sufficient. Future studies should further investigate the application value of artificial intelligence (AI) systems, similar to those being developed for the triage of Barrett’s esophagus and the real-time characterization of colorectal lesions [5,6]. Such systems could be trained to analyze both endoscopic and histopathological images, aiding in the differentiation of RMPS from its mimics by identifying subtle patterns that may elude the human eye.

## Data Availability

The data presented in this study are available on request from the corresponding author due to containing sensitive patient information.
